# Knockdown of IKK1/2 Promotes Differentiation of Mouse Embryonic Stem Cells into Neuroectoderm at the Expense of Mesoderm

**DOI:** 10.1007/s12015-012-9402-7

**Published:** 2012-07-26

**Authors:** Patrick Lüningschrör, Barbara Kaltschmidt, Christian Kaltschmidt

**Affiliations:** 1 Department of Cell Biology, University of Bielefeld, Universitätsstr 25, 33501 Bielefeld, Germany; 2 Molecular Neurobiology, University of Bielefeld, Universitätsstr 25, 33501 Bielefeld, Germany

**Keywords:** NF-κB, Neural crest, Embryonic stem cells, Retinoic acid, Differentiation, Epithelial to mesenchymal transition

## Abstract

Activation of nuclear factor kappa B (NF-κB) is accomplished by a specific kinase complex (IKK-complex), phosphorylating inhibitors of NF-κB (IκB). In embryonic stem cells (ESCs), NF-κB signaling causes loss of pluripotency and promotes differentiation towards a mesodermal phenotype. Here we show that NF-κB signaling is involved in cell fate determination during retinoic acid (RA) mediated differentiation of ESCs. Knockdown of IKK1 and IKK2 promotes differentiation of ESCs into neuroectoderm at the expense of neural crest derived myofibroblasts. Our data indicate that RA is not only able to induce neuronal differentiation in vitro but also drives ESCs into a neural crest cell lineage represented by differentiation towards peripheral neurons and myofibroblasts. The NC is a transiently existing, highly multipotent embryonic cell population generating a wide range of different cell types. During embryonic development the NC gives rise to distinct precursor lineages along the anterior-posterior axis determining differentiation towards specific derivates. Retinoic acid (RA) signaling provides essential instructive cues for patterning the neuroectoderm along the anterior-posterior axis. The demonstration of RA as a sufficient instructive signal for the differentiation of pluripotent cells towards NC and the involvement of NF-κB during this process provides useful information for the generation of specific NC-lineages, which are valuable for studying NC development or disease modeling.

## Introduction

NF-κB is a central regulator of different fundamental cellular processes like apoptosis, cell adhesion, inflammation, immune-response, tissue remodeling and proliferation [1–3]. The mammalian NF-ĸB family consists of five subunits that are able to generate a variety of homo- and hetero- dimeric, DNA-binding complexes. DNA-binding and dimerization is mediated by an N-terminal domain called the Rel-homology domain (RHD) which is shared by all NF-κB subunits. Only the Rel subfamily, p65 (enconded by *relA*), RelB and c-Rel contain a transactivation domain enabling transcriptional activation. In contrast, p50 and p52 lack a transactivation domain. These two subunits cannot activate transcription unless they are partnered to p65, RelB or c-Rel. Furthermore, both p50 and p52 are synthesized from their longer precursors p105 and p100 by proteolytic cleavage [2].

In most unstimulated mammalian cells, NF-ĸB is predominantly located within the cytoplasm bound to its inhibitor, a family of proteins called IĸBs (Inhibitor of kappa B), which mask the nuclear localization signal (NLS) within the RHD domain of the NF-ĸB subunits and prevent translocation of NF-ĸB to the nucleus [2].

Activation of NF-ĸB is obtained by various stimuli which cause degradation of IĸBs, followed by translocation of NF-ĸB to the nucleus and activation of its target genes. Several distinct differences in the activation of NF-κB allow a classification of NF-κB in distinct signaling pathways. Among them, canonical NF-κB signaling is the most common and best characterized NF-κB pathway. During canonical signaling IĸBα is phosphorylated by the IKK complex, which consists of two catalytic subunits IKK1 and IKK2 (IĸB kinases alpha and beta, respectively) and NEMO, a non-catalytic subunit. Upon stimulation the IKK complex is activated, which causes phosphorylation of IĸBα at two N-terminal serine (Ser32 and Ser36) residues by IKK2. Subsequently IĸBα is ubiquitinilated and degraded by the 26S proteasome leading to the translocation of NF-κB to the nucleus and activation of its target genes [2].

Previous studies indicated an upregulation of canonical NF-κB signaling during retinoic acid (RA) mediated differentiation of embryonic stem cells (ESCs) suggesting a potential role of NF-κB during ESC differentiation [4–6]. Recently, it was demonstrated that overexpression of NF-κB partially mimics the effect of RA and causes differentiation of ESCs towards a mesodermal phenotype. Like treatment with RA, canonical NF-κB signaling leads to the induction of Zeb1 and Slug, markers for an epithelial to mesenchymal transition (EMT), as well as to massive changes in the cellular morphology [5]. These data suggest the participation of NF-κB in RA mediated cell fate determination, which is investigated in more detail in this study.

RA is widely used to induce neuronal differentiation of pluripotent cells in vitro [7–9], though in vivo studies indicated that RA signaling is not required for neural induction [10–12]. In the developing mouse embryo RA is not synthesized until E7.5, which is after induction of the neural ectoderm [13]. Furthermore, loss of RA-signaling does not hamper the expression of early neural markers like *Sox1* and *Sox2* [11]. Instead, during early stages of development, RA-signaling is required for further patterning of the posterior neuroectoderm (hindbrain and spinal cord) [11, 12] and a wide range of other developmental processes [14].

Among the transgenic mice with disruptions of enzymes involved in RA synthesis, the strongest phenotype is observed in *Raldh2* knockout mice, which die at E9.5 with an incomplete closure of the neural tube [12]. Other animal-models lacking components of RA-signaling also display severe phenotypes, with defects in NC derivates, among others. Loss of *Rdh10* results in craniofacial defects with an abnormal formation of cranial ganglia and defects in neural crest patterning [15]. Mutant embryos lacking both RALDH1 and RALDH3 show defects in the neural crest derived perioptic mesenchym which is crucial for a proper morphogenesis of the eye [16].

These in vivo studies indicate that RA signaling contributes to the specification of the NC, which takes place during the gastrulation phase at the border between the future neural tube and the epidermis. Later during neurulation, the NC precursors are located within the elevated neural folds and the dorsal parts of the neural tube, from which they delaminate and subsequently migrate. Neural crest cells (NCCs) are highly multipotent and give rise to many different cell types including neurons and glia of the peripheral neurvous system, melanocytes, bone and cartilage cells and smooth muscle cells.

Recent studies indicated that some NCCs still persist in adulthood, especially in a number of craniofacial tissues [17–19]. Their high regenerative potential and high degree of multipotency makes neural crest derived adult stem cells a valuable source for adult stem cells with potential application for regenerative medicine [20].

Other in vitro studies using pluripotent stem cells also suggested that RA-signaling is implicated in the directed differentiation of pluripotent stem cells towards NCCs [21–23], providing a desirable tool for studying NC development, cell fate determination and migration. NCCs derived from patient-specific induced pluripotent stem cell (iPSCs) even enable modeling of human diseases [24].

In this study we report that treatment of mouse ESCs with RA is sufficient to induce differentiation towards a NC like phenotype, whose further specification depends on NF-κB signaling, which has not been described yet. Our data provide new insight into the mechanism of NC specification and might be helpful for the directed differentiation or even programming of other cell populations towards specific NC lineages with a defined identity along the anterior-posterior axis.

## Results

### Generation of Neural Crest Precursors from Embryonic Stem Cells by Treatment with RA

Previous reports showed that RA signaling is able to induce a neural differentiation in vitro [7–9]. The impact of RA on the generation of NCCs remains quite elusive with only a few studies available addressing this issue [21, 22].

**Fig. 1:**
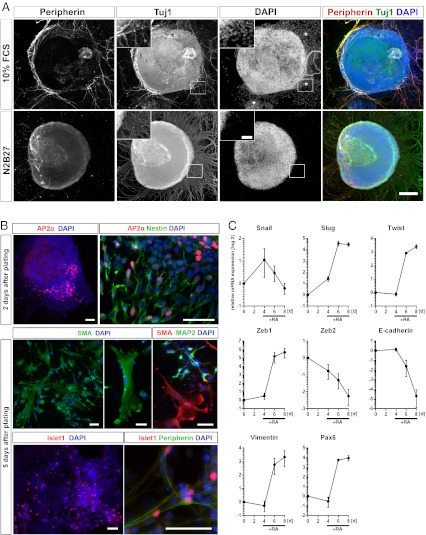
Retinoic acid promotes expression of markers associated with an EMT during ESC differentiation. (**a**) Peripherin and Tuj1 stainings of EBs 5 days after plating in media containing either 10 % FCS or N2 and B27. Asterisks indicate migrating cell populations. Scale bar: 200 μm. Scale bar, All insets: 50 μm (**b**) EBs were plated in medium containing 10 % FCS and immunohistochemically analyzed 2 days and 5 days after plating. Negative data for Otx2 are not shown. Scale bars: 50 μm. (**c**) qPCR analysis of different EMT and NC markers during RA induced differentiation of ESCs. Data indicate mean ± SD of one experiment performed as triplicate representing at least two independent experiments. In all experiments RA was applied after 4 and 6 days of EB formation. For immunohistochemical analysis in (**a**) and (**b**) EBs were plated 8 days after formation and further cultivated as indicated

To study the effect of RA during ESC differentiation, cells were cultivated for 8 days in suspension as embryoid bodies (EBs) without LIF and RA was applied at the 4th and 6th day of differentiation. After 8 days, EBs were either plated in EB-medium containing 10 % FCS or serum-free N2B27 medium and stained for Tuj1 and Peripherin. Tuj1 was used as a general marker for neurons, whereas Peripherin only labels the subclass of peripheral neurons. In contrast to the effect of N2B27 medium, which yielded a large amount of Tuj1^+^ cells with only a poor overlap to Peripherin (Fig. 1a), plating of EBs in the presence of serum resulted in a fewer number of Tuj1^+^ cells, with a high amount of Tuj1^+^/Peripherin^+^ cells. We also observed a large number of migrating cells in the presence of serum, which were hardly detectable in N2B27 medium.

To further investigate the effect of serum on the resulting phenotype, cells were stained for the neural crest marker AP2α to examine the presence of NCCs. AP2α^+^ as well as AP2α^+^/Nestin^+^ cells appeared predominantly as a migrating cell population at the border of plated EBs (Fig. 1b). 5 days after further cultivation we were able to detect SMA^+^ cells and MAP2^+^ cells indicating the presence of both, the mesodermal and ectodermal lineage. To specify the identity of the neuronal cells more detailed, antibodies for Islet1, Otx2 and Peripherin were applied (Fig. 1b). The presence of Islet1^+^ cells as well as of Islet1^+^/Peripherin^+^ cells indicates NC derived peripheral neurons with a dorsal root ganglia (DRG) identity (Fig. 1b). In contrast, Otx2^+^ cells, which indicate progenitors with a forebrain and midbrain identity, were not detectable reflecting the caudalizing effect of RA.

Our data so far suggest that RA treatment results in a NC like intermediate which is able to differentiate into cells of the PNS as well as SMA^+^ myofibroblasts in the presence of serum. It is not likely, that the obtained SMA^+^ cells are directly derived by mesodermal precursors since RA inhibits mesodermal differentiation [21].

During delamination from the neural tube, NCCs undergo an epithelial to mesenchymal transition (EMT), which involves massive cytoskeletal rearrangements and morphological changes towards a motile cell phenotype enabling migration and specification of their different derivates [25, 26]. This is an essential process for the generation and specification of the NC and their derivates during development.

To examine the effect of RA at the transcriptional level, expression of different EMT and NC markers was investigated using qPCR. Treatment with RA resulted in an induction of NC and EMT markers *Slug* (*snai2*), *Twist* (*twist1*), *Vimentin* (*vim*), *Zeb1*, and *Pax6* whereas *E-cadherin* (*cdh1*) expression markedly decreased upon RA treatment. mRNA levels of *Snail* (*Snai1*) seemed not to be upregulated upon RA treatment (Fig. 1c), though Snail is one of the major inductors of an EMT. The second member of the Zeb family, *Zeb2*, was slightly downregulated after treatment with RA (Fig. 1c).

The induction of several key EMT- and NC markers after RA treatment indicates that RA not only induces the differentiation towards a neuroepithelium by activating *Pax6* expression, but also promotes an upregulation of some EMT markers at the transcriptional level which might result in the generation of a NC like phenotpye.

**Fig. 2:**
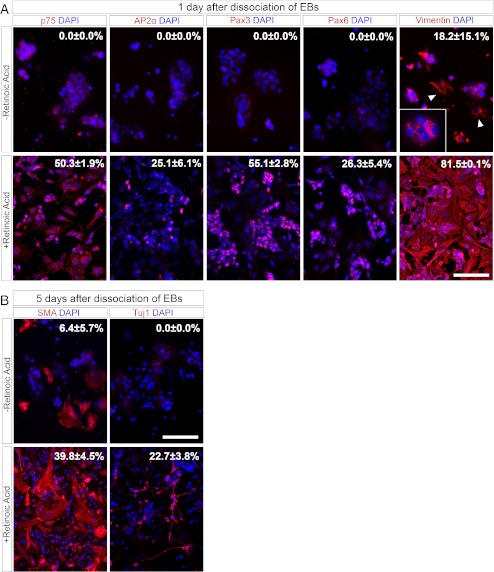
Neural crest related markers are expressed upon retinoic acid mediated differentiation of ESCs. (**a**) RA treated or untreated EBs were immunohistochemically analysed for p75, AP2α, Pax3, Pax6 and Vimentin 1 day after dissociation. Though in untreated cells Vimentin expression was detectable in form of non-filamentous cluster (inset), Vimentin filaments were only detectable in the minority of the cells (indicated by *arrowheads*). Scale bar: 100 μm. (**b**) 5 days after dissociation of RA treated or untreated EBs, cells were labeled for SMA and Tuj1. Scale bar: 200 μm. RA was applied after 4 and 6 days of EB formation as indicated. Dissociation of EBs was performed after 8 days of cultivation in suspension. For quantification of immunohistochemical analysis >300 cells were evaluated. Data indicate Mean ± SD

To further characterize the effect of RA during ESC differentiation the expression of several markers was compared between RA treated cells and untreated controls. Four days after EB-formation, cells were either treated for 4 days with RA or left untreated. In order to quantify the expression pattern, EBs were dissociated to single cells and stained for the markers indicated (Fig. 2).

One day after dissociation 25.1 % and 55.1 % of the RA-treated cells were positive for the transcriptions factors Ap2α and Pax3, respectively, whereas 50.3 % were positive for p75. To investigate the presence of NE precursors, dissociated EBs were also stained for Pax6, which was expressed in 28.3 % of the RA-treated cells (Fig. 2a). None of the markers mentioned above could be detected in untreated controls, indicating that these cells rather represent an undifferentiated phenotype (Fig. 2a).

After dissociation untreated cells also displayed a more densely packed morphology and tended to grow in patches, in contrast to RA treated cells, which showed a more even distribution with mostly a wide spread morphology. This was also reflected by the expression of the intermediary filament Vimentin. 81.5 % of the RA treated cells showed a cytoskeleton built from Vimentin, whereas in untreated cells Vimentin filaments were only detectable to a lesser extent (18.2 %) (Fig. 2a).

Five days after plating 22.68 % of the cells treated with RA were TUJ1^+^ and 39.82 % showed an expression of SMA (Fig. 2b). In contrast only 6.4 % of the untreated controls expressed SMA and none of the cells left untreated were TUJ1^+^ (Fig. 2b).

Though our data do not demonstrate the generation of bona fide NCCs by RA treatment of ESCs, they provide strong evidence that RA promotes the expression of several NC markers as well as the expression of some markers for an EMT. Additionally, the difference in the cellular morphology between RA treated cells and untreated controls further indicate that RA participates in promoting an EMT, resulting in a Vimentin^+^, NC like phenotype.

The presence of cells positive for NC markers as well as Pax6^+^ cells indicates a mixed population of precursor cells, which enables the generation of both the mesodermal and neueoectodermal lineage during maturation of the progenitor cells in the presence of serum.

### Inhibition of NF-κB Signaling Promotes Neuronal Differentiation at the Expense of Mesoderm

**Fig. 3:**
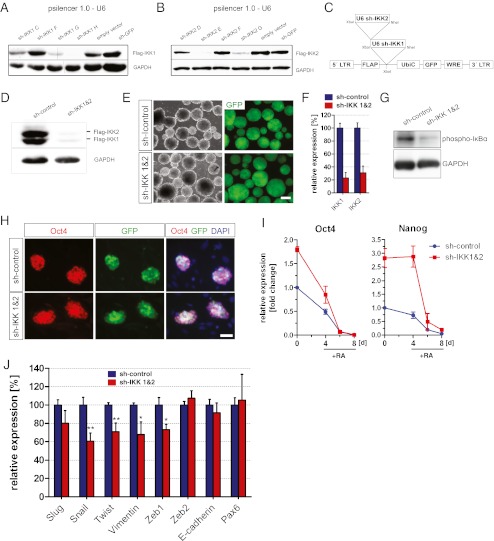
Knockdown of IKK1 and IKK2 in ESCs. (**a, b**) Western blots were carried out after co-expression of flag-tagged IKK1 (**a**) or IKK2 (**b**) with the indicated sh-sequences in HEK 293FT cells. (**c**) Lentiviral vector for inhibition of NF-κB signaling by knockdown of IKK1 and IKK2. LTR, long terminal repeat. WRE, woodchuck post-transcriptional regulatory element. hUbiQ, human Ubiquitin C promoter. U6, murine U6 promoter. (**d**) Validation of the lentiviral vector. Western blots were performed after ectopic co-expression of Flag-tagged IKK1 and IKK2 together with the lentiviral vector in HEK 293FT cells. (**e**) J1 ESCs, transduced with the lentiviral vectors indicated, were differentiated as EBs. Scale bar: 200 μm. (**f**) To validate the knockdown of IKK1 and IKK2, qPCR were carried out after differentiation of ESCs as EBs. Data represent mean ± SEM of three independent experiments, each performed as triplicate. (**g**) Knockdown of IKK1 and IKK2 results in reduced levels of phosphorylated IκBα. ESCs were differentiated for 8 days as EBs and treated with RA after 4 and 6 days. Proteins were extracted after 8 days and Western blots were performed. (**h**) Oct4 stainings of transduced ESCs to confirm maintenance of pluripotency after knockdown of IKK1 and IKK2. Feeder cells were also stained by DAPI, but negative for Oct4 and GFP. Scale bar: 50 μm. (**i**) Expression levels of *oct4* and *nanog* during differentiation of ESCs as EBs. After 4 days cells were treated with RA. Data indicate mean ± SD of one experiment performed as triplicate representing two independent experiments. (**j**) Effect of NF-κB inhibition assayed by qPCR. After transduction of J1 ESCs, cells were differentiated as EBs and treated for 4 days with RA. At the day of EB dissociation samples were collected and different markers were assayed. Data represent mean ± SEM of three independent experiments, each performed as triplicate

Recent studies suggested a role for NF-κB signaling during differentiation of ESCs, indicating that NF-κB is able to promote an EMT of ESCs which causes loss of pluripotency [5, 6]. To explore the impact of NF-κB during the process of cell fate determination, we generated a lentivirus-based vector to deliver two U6-promoter driven short-hairpin RNA (shRNA) cassettes producing a simultaneous knockdown of both IKKs (inhibitor of NF-kappa B kinases) (Fig. 3c).

NF-κB belongs to an inducible family of transcription factors, whose activation relies on phosporylation of its inhibitor, a protein family called IκBs (inhibitor of NF-kappa B). The IKK complex, consisting of IKK1, IKK2 and a non-catalytic subunit NEMO, is necessary for activation of NF-κB signaling and results in phosphorylation and subsequent degradation of IκB. Simultaneous knockdown of both IKKs therefore does not restrict the inhibition of NF-κB to one subunit.

Different shRNAs were tested for their potential to reduce the protein levels of IKK1 and IKK2 in HEK 293FT cells, respectively. Flag-tagged IKK1 or IKK2 were co-expressed together with each shRNA (Fig. 3a and b) and for each kinase a functional sequence was selected and a “double-knockdown” vector was generated (Fig. 3c and d). Two non-functional sequences served as control.

After transduction, ESCs were expanded as a polyclonal cell-line and subsequently differentiated as EBs (Fig. 3e). For validation of a successful knockdown of IKK1 and IKK2, the expression level of both kinases was assayed by qPCR after 8 days of differentiation. In both cases a reduction in the mRNA expression could be detected (Fig 3f). The effect of reduced IKK levels on NF-κB signaling was also investigated after ESC differentiation. Since the IKK-complex phosphorylates IκBα, which is subsequently degraded by the proteasome and thereby causes an activation of NF-κB, we investigated the phosphorylation of IκBα. After 8 days of differentiation as EBs and treatment with RA, reduced levels of phosphorylated IκBα could be observed upon knockdown of both IKKs (Fig 3g), indicating a functional impairment of NF-κB signaling.

To confirm the maintenance of pluripotency after transduction of ESCs and knockdown of IKK1 and IKK2, Oct4 stainings were carried out (Fig. 3f). ESCs expressing either both functional sh-RNAs or the corresponding control sh-RNAs were positive for Oct4, demonstrating that knockdown of IKK1 and IKK2 had no obvious effect on self-renewal and pluripotency of ESCs (Fig. 3f). For further characterization of knockdown ESCs, the expression of *oct4* and *nanog* was monitored during RA induced differentiation by qPCR (Fig. 3g). Expression of both pluripotency markers was slightly enhanced in the undifferentiated state and the downregulation of *oct4* and *nanog* was delayed during the first days of differentiation upon LIF withdrawal. Nevertheless, knockdown of IKK1 and IKK2 did not mediate any resistance to RA induced ESC differentiation. Treatment with RA caused a dramatic drop in the expression of both pluripotency markers (Fig. 3g).

These observations confirm previous data demonstrating that overexpression of a dominant negative form of IκBα caused a slight delay in the expression of Nanog after LIF withdrawal [6]. This is in agreement with the notion that NF-κB signaling is repressed during pluripotency and upregulated upon differentiation [5, 6].

To investigate the effect of inhibiting NF-κB signaling during RA induced differentiation, *Pax6* and different markers characteristic for an EMT were assayed by qPCR as well. A significant downregulation in the expression of *Snail*, *Twist*, *Vimentin* and *Zeb1* could be detected (Fig. 3f). Previous studies already demonstrated a NF-κB dependent regulation of these genes, indicating a reduced transcriptional activity of κB-targets after knockdown of IKK1 and IKK2.

**Fig. 4:**
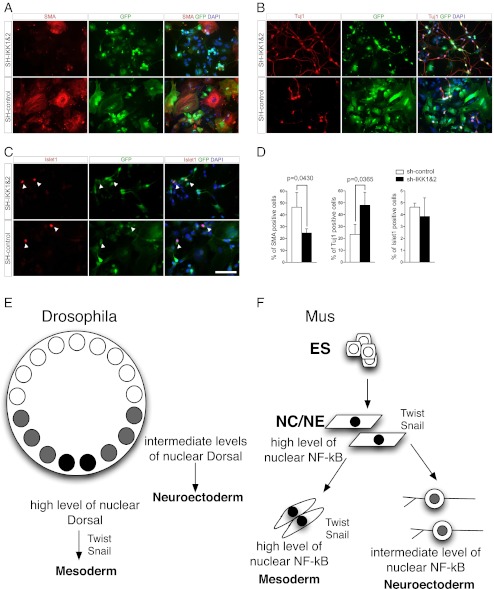
NF-κB promotes RA mediated differentiation of ESCs into neuroectoderm at the expense of mesoderm. (**a–c**) Four days after formation of EBs, cells were treated for 4 days with RA and dissociated after 8 days of differentiation. Five days after dissociation of EBs and replating, cells were stained for SMA (**a**), Tuj1 (**b**) and Islet1 (**c**). Scale bar: 200 μm. (**d**) Quantification of immunostainings. Only GFP^+^ cells were evaluated. Data show mean ± SD of three independent experiments; >750 cells were quantified. (**e**) Scheme of a cross-sectioned *Drosophila* embryo. Nuclei within the syncytium are either with high level of nuclear Dorsal (*black, ventral side*), intermediate levels of nuclear Dorsal (*grey*) or with no nuclear dorsal (*white*). High levels of dorsal activate target genes *Twist* and *Snail,* necessary for mesoderm formation, whereas neuroectoderm is formed with intermediate levels of dorsal. (**f**) Hypothetical pathway of cell fate induction by NF-κB in mESCs. ESCs are devoid of translated NF-κB [4]. Forced expression of NF-κB resulted in the formation of mesoderm [4], whereas this study indicates that knockdown of IKK1 and IKK2 enhanced the formation of neuroectoderm. ES: Embryonic stem cells; NC: Neural crest cells; NE: Neuroepithelial cells

Next, the effect on cell fate determination during RA mediated differentiation of ESCs was investigated. Five days after dissociation of EBs cells were stained for SMA, Tuj1and Islet1 (Fig. 4a, b and c) and the number of positive cells was quantified (Fig. 4d). To measure the effects of sh-RNA expression only, GFP^+^ cells were evaluated. Upon NF-κB inhibition a shift from SMA^+^ cells towards Tuj1^+^ cells as compared to the control was observed. The number of SMA^+^ cells decreased from 46.37 % to 24.66 %, whereas the number of Tuj1^+^ cells increased from 23.41 % to 47.96 % (Fig. 4c). In contrast, significant differences in the number of Islet1^+^ cells were not detectable (Fig. 4d). The remaining cells that were neither positive for Tuj1 nor SMA, might represent a mixture of undifferentiated precursors, differentiated cells of another phenotype or ESCs that still remained pluripotent and were not further investigated.

In summary, we demonstrate in accordance with [22] that RA-treatment can be used to generate NCCs from ESCs. An increase of the amount of neuronal cells at the expense of a mesodermal phenotype was detectable upon inhibition NF-κB signaling, indicating a role for NF-κB signaling in cell fate determination during RA-induced differentiation of pluripotent cells.

## Discussion

Understanding the mechanisms underlying the specification of the NC provides essential information for the generation of lineage specific NC-precursors with a defined identity along the anterior-posterior axis. Our study demonstrates that RA-signaling is one of the pathways triggering the induction of NC identity. Further investigations might reveal, whether the generation of RA-treatment directly triggers the differentiation of ESCs into NCCs or if RA rather induces a neuroepithelial phenotype and derivation of NCCs is a secondary effect promoted by intrinsic cues resembling the in vivo situation with a NC specification at the border between neuroectoderm and non-neural ectoderm. The fact that treatment of EBs with RA creates a gradient, with the highest RA concentration at the outside, indicates that only those cells exposed to certain RA-concentrations generate NC.

Indeed, previous reports using Sox10-GFP reporter cell-line already suggested that only specific concentration of RA are able to induce a NC cell fate [22]. Further investigation and manipulation of other signaling pathways in addition to different RA concentrations may provide further details for the specification of NC and the generation of stabile NC precursors.

Our data also suggest a role for NF-κB during this process. In vitro, inhibition of NF-κB signaling during RA induced ESC differentiation resulted in a shift from a mesodermal phenotype towards an ectodermal one. Inhibition of NF-κB signaling seems to interfere with the specification of ESCs towards a SMA^+^ phenotype resulting in an increased amount of Tuj1^+^ cells.

There is either the possibility that reduced activation of NF-κB acts during early cell specification, resulting in an enhanced number of NE precursors or NF-κB activation is necessary for the specification of NC derived SMA^+^ cells in later stages of differentiation. The unaltered amount of NC derived Islet1^+^ cells might indicate that low levels of activated NF-κB results in an increased amount of NE precursors, favoring the first possibility. Furthermore, previous studies already demonstrated that NF-κB signaling is already upregulated 1 day after treatment with RA, which also indicates that NF-κB rather act during initial differentiation steps [6]. We also observed a downregulation of different EMT markers upon inhibition of NF-κB signaling, also supporting the notion that dysregulated NF-κB signaling effects the specification of the precursor due to reduced transcriptional activity of κB-targets. A more detailed analysis using linage tracing or inducible promoters to repress NF-κB signaling might address this question and would provide further insight into the effect of NF-κB signaling on cell fate specification in vitro*.*

In vivo, transgenic animals with disruptions of single NF-κB subunits show no developmental defects [27, 28], but mice double deficient for *IKK1* and *IKK2* [29] as well as knockout of *Bcl10*, a receptor interacting protein which is crucial for full activation of IKKs, show a neural tube closure defect [30]. Notably, such a defect is also observed in knockout mice for *Ap2α*, *Pax3* and *Pax7*, factors which are essential for the specification of the neural crest [31, 32]. In agreement with the notion that NF-κB is a positive regulator for *Twist* and *Snail* [33], knockout of *Twist1* results in strong developmental defects of neural crest derivates [33], whereas in *Snail* deficient mice the mesoderm retains an epithelial characteristic. This demonstrates the importance of Snail in inducing an EMT [34], which is crucial for the generation of the neural crest.

The *Drosophila* orthologue of NF-κB, *dorsal*, is essential for patterning the dorsal-ventral axis during development [35]. Briefly, high levels of Dorsal determine the presumptive mesoderm by activating *Snail* and *Twist*, whereas lower levels result in neuroectoderm [36] (Fig. 4e). Since Bilateria show a common plan for patterning the dorsoventral axis [37], in mammals, a NF-κB gradient might be necessary for establishing the neural crest. Here we show that knockdown of IKK1/2 reduces NF-κB target gene expression (*Twist*, *Snail*). This might result in an intermediate level of NF-κB activation, promoting the formation of neuroectoderm (Fig. 4f). In accordance with this model, we hypothesize that knockdown of IKK1/2 reduces differentiation towards NCCs, a cell populations with high NF-κB activity. We conclude that a *Drosophila* gene cascade of the *dorsal* complex might have a similar function in mammalian development.

## Material & Methods

### Cell Culture, Differentiation & Transfection

J1 ESCs were cultured on Mitomycin C inactivated mouse embryonic fibroblasts (MEFs) in ESC medium consisting of Dulbeccos-Minimum Essential Medium (DMEM; PAA) supplemented with 15 % fetal calf serum (FCS; Gibco), 1,000 U/ml LIF (Millipore), 1× non-essential amino acids (PAA), 2 mM L-glutamine (PAA), 1× Penicillin/Streptomycin (PAA) and 150 μM β-mercaptoethanol.

For differentiation, ESCs were cultured in suspension as EBs in EB-medium and treated with retinoic acid (5 μm, Sigma-Aldrich) at the 4th and 6th day of differentiation. EB-medium consisted of DMEM containing 10 % FCS (Gibco), 1× non-essential amino acids (PAA), 2 mM L-glutamin (PAA), 1× Penicillin/Streptomycin (PAA) and β-mercaptoethanol.

After 8 days EBs were either plated in EB-medium or serum-free N2B27-medium consisting of a 1:1 mix of Neurobasal (Invitrogen) and DMEM/F12 (Invitrogen) media supplemented with 0.5× N2 (Invitrogen), 0.5× B27 (Invitrogen), 1× non-essential amino acids (PAA), 2 mM L-glutamine (PAA), 1× Penicillin/Streptomycin (PAA).

HEK 293FT cells were cultivated in DMEM containing 10 % FCS and transfected with Turbofect (Fermentas) according to the manufactures’ instructions. For co-expression of flag-tagged IKK1 and IKK2 (600 ng) with each sh-expression vector (2,400 ng), 1 × 10^6^ cells per cm^2^ were plated on 6-well plates and directly transfected after plating. In all transfections, 3 μl Turbofect was used and the total amount of DNA was kept constant by addition of the according empty vector.

### RT-PCR and qPCR

For quantification of mRNA total RNA was prepared using RNAeasy RNA Kit (Qiagen). 1 μg of total RNA was treated with DNAse I (Fermentas) and subsequently reverse transcribed with First strand cDNA synthesis Kit (Fermentas). 1 μl of 1:5 diluted cDNA was used as template per reaction. Each reaction was performed as triplicate.

All qPCR reactions were performed using Platinum SYBR Green qPCR Super-Mix UDG (Invitrogen) and assayed with a Rotor Gene 6000 (Corbett).

### Western Blot

Cells were extracted in lysis-buffer (1 % SDS, 5 mM EDTA, complete protease inhibitor cocktail (Roche)) directly in the dish on ice. Subsequently extracts were boiled for 5 min at 95 °C. Equal amounts of protein extracts were separated on a 10 % SDS-PAGE and transferred to nitrocellulose membranes (PALL). Membranes were blocked in PBS with 5 % milk powder and 0,05 % Tween for 1 h at 37 °C. Blots were probed with primary antibodies overnight and incubated with horseradish peroxidase-conjugated secondary antibodies the next day for 1 h. Western blots were carried out using GAPDH (Santa Cruz, sc-32233), pospho IκBα (Ser 32/36) (Cell Signaling, 5A5) and Flag (Sigma Aldrich; F7425) primary antibodies and developed using ECL (enhanced chemiluminescence).

### Lentivirus Production

Lentivirus production was perfomed by co-transfection of 293FT cells using calcium-phosphate precipitation. One day before transfection 1 × 10^7^ cells were plated on a 15 cm dish. The next day cells were transfected with 50 μg of the transfer vector indicated, 37,5 μg Δ8.91 and 15 μg VSV-G helper plasmids. 16–24 h later medium was changed and 60–72 h after transfection the supernatant was harvested and stored at −80 °C or used immediately for concentration by ultracentrifugation (50.000 g, 2 h, 4 °C).

### Immunostainings

Cells were fixed with 4 % paraformaldehyde in PBS for 15 min at room temperature, permeabilized for 10 min with 0,1 % Triton-X-100 in PBS, blocked for 45 min in 2 % bovine serum albumin (BSA) and incubated with the primary antibody for 2 h at room temperature. Incubation with the appropriate Alexa-conjugated secondary antibody (Invitrogen) was performed at room temperature for 1 h. DAPI was used as counterstain. Immunostainings were carried out with Pax3 (DSHB), Pax6 (DSHB), p75 (Millipore; AB1554), AP2α (DSHB; 3B5), Islet11 (DSHB; 40.2D.6), Otx1 (DSHB; 5F5), MAP2 (Millipore; AB5622), Tuj1 (Promega; 5G8), Smooth Muscle Actin (SMA) (Sigma-Aldrich; 1A4), Peripherin (Millipore, AB 1530) and Vimentin (DSHB, 40E-C) primary antibodies.

### Plasmid Construction

To generate IKK1 and IKK2 knockdown plasmids, different sh-sequences were synthesized as sense and antisense oligos and cloned into ApaI and EcoRI restriction sites of psilencer U6-1.0. A unique NheI restriction site was introduced at the 3′ end of the oligos. After validation of different sequences, one functional and non-functional sequence was selected for each kinase and subsequently introduced into FG12 [38]. Following sequences were used for the simultaneous knockdown: IKK1; 5′ CAGCCTTTGTAGTTTAATA 3′ (sequence H), IKK2; 5′ GGACATCGTTGTTAGTGAA 3′ (sequence E). As a control, two non-functional sequences were used: IKK1; 5′ GCATGAATGTGTCTCGACT 3′ (sequence F), IKK2; 5′ TGACGTGAAGCATCTAGTA 3′ (sequence D). An XbaI and NheI fragment, consisting of U6 promoter, sh-sequence and termination signal was cloned into the XbaI restriction site of FG12. During introduction of the first sh-cassette the NheI restriction site was destroyed and the XbaI restriction site could be used again, for introduction of the second sh-cassette.
